# Host Range Determinants of *Pseudomonas savastanoi* Pathovars of Woody Hosts Revealed by Comparative Genomics and Cross-Pathogenicity Tests

**DOI:** 10.3389/fpls.2020.00973

**Published:** 2020-07-02

**Authors:** Alba Moreno-Pérez, Adrián Pintado, Jesús Murillo, Eloy Caballo-Ponce, Stefania Tegli, Chiaraluce Moretti, Pablo Rodríguez-Palenzuela, Cayo Ramos

**Affiliations:** ^1^ Área de Genética, Facultad de Ciencias, Universidad de Málaga, Málaga, Spain; ^2^ Instituto de Hortofruticultura Subtropical y Mediterránea “La Mayora”, Consejo Superior de Investigaciones Científicas (IHSM-UMA-CSIC), Málaga, Spain; ^3^ Institute for Multidisciplinary Research in Applied Biology, Universidad Pública de Navarra, Mutilva Baja, Spain; ^4^ Dipartimento di Scienze e Tecnologie Agrarie, Alimentari Ambientali e Forestali (DAGRI), Laboratorio di Patologia Vegetale Molecolare, University of Florence, Firenze, Italy; ^5^ Department of Agricultural, Food and Environmental Science, University of Perugia, Perugia, Italy; ^6^ Centro de Biotecnología y Genómica de Plantas, Universidad Politécnica de Madrid (UPM)-Instituto Nacional de Investigación y Tecnología Agraria y Alimentaria (INIA), Madrid, Spain; ^7^ Departamento de Biotecnología-Biología Vegetal, Escuela Técnica Superior de Ingeniería Agronómica, Alimentaria y de Biosistemas, UPM, Madrid, Spain

**Keywords:** *Pseudomonas syringae*, *Pseudomonas savastanoi* pathovars, woody hosts, host range, virulence factors, comparative genomics, type III effectors, phytotoxins

## Abstract

The study of host range determinants within the *Pseudomonas syringae* complex is gaining renewed attention due to its widespread distribution in non-agricultural environments, evidence of large variability in intra-pathovar host range, and the emergence of new epidemic diseases. This requires the establishment of appropriate model pathosystems facilitating integration of phenotypic, genomic and evolutionary data. *Pseudomonas savastanoi* pv. savastanoi is a model pathogen of the olive tree, and here we report a closed genome of strain NCPPB 3335, plus draft genome sequences of three strains isolated from oleander (pv. nerii), ash (pv. fraxini) and broom plants (pv. retacarpa). We then conducted a comparative genomic analysis of these four new genomes plus 16 publicly available genomes, representing 20 strains of these four *P. savastanoi* pathovars of woody hosts. Despite overlapping host ranges, cross-pathogenicity tests using four plant hosts clearly separated these pathovars and lead to pathovar reassignment of two strains. Critically, these functional assays were pivotal to reconcile phylogeny with host range and to define pathovar-specific genes repertoires. We report a pan-genome of 7,953 ortholog gene families and a total of 45 type III secretion system effector genes, including 24 core genes, four genes exclusive of pv. retacarpa and several genes encoding pathovar-specific truncations. Noticeably, the four pathovars corresponded with well-defined genetic lineages, with core genome phylogeny and hierarchical clustering of effector genes closely correlating with pathogenic specialization. Knot-inducing pathovars encode genes absent in the canker-inducing pv. fraxini, such as those related to indole acetic acid, cytokinins, rhizobitoxine, and a bacteriophytochrome. Other pathovar-exclusive genes encode type I, type II, type IV, and type VI secretion system proteins, the phytotoxine phevamine A, a siderophore, c-di-GMP-related proteins, methyl chemotaxis proteins, and a broad collection of transcriptional regulators and transporters of eight different superfamilies. Our combination of pathogenicity analyses and genomics tools allowed us to correctly assign strains to pathovars and to propose a repertoire of host range-related genes in the *P. syringae* complex.

## Introduction

The *Pseudomonas syringae* species complex is considered the most relevant phytopathogenic bacterium worldwide, as a prominent research model and as an economically important phytopathogen (Mansfield et al., 2012). It comprises at least 13 phylogroups (PGs) encompassing 15 different *Pseudomonas* species associated with plants and the water cycle (Berge et al., 2014; Gomila et al., 2017). The complex is also divided at an infrasubspecific level into ca. 65 pathovars defined by characteristic plant host ranges (Bull and Koike, 2015).

The very broad host range of the *P. syringae* complex contrasts with the host specialization of most individual isolates from agricultural crops. Conversely, environmental isolates show a wider host range (Morris et al., 2013; Berge et al., 2014; Monteil et al., 2016). Moreover, a recent study showed that *P. syringae* strains form an overlapping continuum of host range potential and that pathovar denominations do not correspond to the *P. syringae* biology (Morris et al., 2019). Understanding this complexity at the different levels requires the establishment of appropriate pathosystems, facilitating integration of phenotypic, genomic, and evolutionary data. Consequently, the *P. syringae* complex emerged as one of the most relevant models to study host specificity in bacterial phytopathogens (Vinatzer et al., 2014; Baltrus et al., 2017; Dillon et al., 2019b).

*Pseudomonas savastanoi* belongs to PG3 of the *P. syringae* complex. This is the only phylogroup including bacteria causing tumorous overgrowths (knots) in woody hosts, namely *P. savastanoi* pv. savastanoi (Psv), *P. savastanoi* pv. nerii (Psn) and *P. savastanoi* pv. retacarpa (Psr), *P. meliae*, *P. tremae,* and *P. syringae* pathovars cerasicola, daphniphylli, dendropanacis, myricae, and rhaphiolepidis (Lamichhane et al., 2014; Caballo-Ponce et al., 2017a). Additionally, isolates of *P. savastanoi* pv. fraxini (Psf) cause cankers accompanied by excrescences in ash (*Fraxinus excelsior*) (Janse, 1981; Gardan et al., 1992; Caballo-Ponce et al., 2017a). It was experimentally determined through pathogenicity tests that these four pathovars have different host ranges, namely: Psv induces knots in olive (*Olea europaea*) and ash but not in oleander (*Nerium oleander*); Psn in all three hosts; and Psr only in broom (*Retama sphaerocarpa*) (Álvarez et al., 1998). In turn, Psf induces symptoms both in ash and olive (Janse, 1982; Iacobellis et al., 1998; Ramos et al., 2012). Nevertheless, some *P. savastanoi* populations show heterogeneous host ranges, which can even drastically differ between strains of the same pathovar (Cinelli et al., 2014; Morris et al., 2019). At the same time, and likely because of the reported lack of genetic diversity and overlapping host ranges, various genetic and phenotypical analyses did not establish a clear-cut differentiation among these pathovars (Janse, 1991; Sisto et al., 2007; Cinelli et al., 2014; Moretti et al., 2017). Thus, this well-established delineation of the host range, converts these four *P. savastanoi* pathovars of woody hosts in an excellent system to study the basis of host range in knot- and canker-forming bacterial pathogens.

Here, we performed comparative genomics analyses of 20 P*. savastanoi* strains isolated from olive, oleander, ash, and broom, covering all four well-defined *P. savastanoi* pathovars of woody hosts. First, we obtained the complete chromosome sequence of Psv NCPPB 3335 and draft genome sequences of Psn *Psn23*, Psr CECT 4861 and Psf NCPPB 1006. Second, cross-pathogenicity tests allowed correlating the results of the genomics analyses with host specificity. We thus delineated the core and pan-genome of the four *P. savastanoi* pathovars of woody hosts and identified the set of pathovar- and strain-specific gene complements. Our results provide a collection of putative virulence genes that can now be experimentally tested for their contribution to the definition of host specificity in knot- and canker-forming bacterial pathogens and their evolution into pathovars.

## Materials and Methods

### Bacterial Strains and Growth Conditions

*P. savastanoi* (**Table 1**) were routinely grown at 28°C in lysogeny broth (LB) medium (Bertani, 1951) without glucose and containing 0.5% NaCl. When required, media were supplemented with (final concentrations) nitrofurantoin (Nf, 20 µg/ml) and cycloheximide (Ch, 100 µg/ml).

**Table 1:** General genome features of *Pseudomonas savastanoi* strains isolated from woody host.

Pathovar/Strain^a^	Genome size (Mb)	G+C content (%)	Number of genes	Protein-coding sequences	Contigs	Coverage	Accession number^d^	Reference
**pv. fraxini**								
NCPPB 1006	5.89	58.1	5584	5054	222	250×	NZ_NIAW00000000	This study
ICMP 7711^PT^ ^b^	6.02	57.9	5784	5155	481	68×	NZ_LLJL00000000	Thakur et al., 2016
CFBP 5062^PT^ ^b^	6.26	58.0	5828	5430	330	78×	NZ_LIIC00000000	Nowell et al., 2016
ICMP 9132	6.05	58.0	5766	5228	575	90.9×	NZ_RBSB00000000	Dillon et al., 2019b
ICMP 7712	5.92	58.1	5583	5091	686	75.8×	NZ_RBSC00000000	Dillon et al., 2019b
ICMP 9129	5.99	58.1	5692	5163	588	105.1×	NZ_RBSA00000000	Dillon et al., 2019b
**pv. nerii**								
*Psn23*	5.84	58.1	5568	5018	229	250×	NZ_NIAY00000000	This study
ICMP 16943^PT^ ^b^	5.72	58.3	5465	4874	341	45.2×	NZ_LJQW00000000	Thakur et al., 2016
CFBP 5067^PT^ ^b^	5.79	58.2	5602	4965	242	54×	NZ_LIHX00000000	Nowell et al., 2016
ICMP 13781	6.05	57.9	5658	5658	811	35.7×	RBTO00000000	Dillon et al., 2019b
ICMP 16944	5.80	58.1	5479	4944	532	53.3×	NZ_RBUB00000000	Dillon et al., 2019b
**pv. retacarpa**								
CECT 4861^PT^ ^b^	5.73	58.1	5516	4920	316	250×	NZ_NBYW00000000	This study
ICMP 16945^PT^ ^b^	5.67	58.1	5457	4686	510	45.3×	NZ_LJRD00000000	Thakur et al., 2016
ICMP 16946	5.72	57.9	5373	5373	1103	30.6×	RBQM00000000	Dillon et al., 2019b
ICMP 16947	5.79	58.1	5648	4972	564	46.5×	NZ_RBNM00000000	Dillon et al., 2019b
**pv. savastanoi**								
NCPPB 3335^c^	6.17	58.0	5916	5291	4	200×	NZ_CP008742 FR820585 FR820586 FR820587	This study
								Bardaji et al., 2011
ICMP 4352^PT^	6.02	58.0	5773	5215	321	66.9×	NZ_LJRJ00000000	Thakur et al., 2016
DAPP-PG722	6.42	57.9	6309	5545	412	70×	NZ_JOJV00000000	Moretti et al., 2014
PseNe107	6.07	58.0	5793	5263	247	140×	NZ_JYHF00000000	Bartoli et al., 2015
ICMP 13519	6.26	57.9	6168	5507	426	90.6×	NZ_RBNW00000000	Dillon et al., 2019b
ICMP 1411	5.80	58.1	5680	5058	383	49.7×	NZ_RBPF00000000	Dillon et al., 2019b
0485_9*	5.94	57.9	5946	5946	1918	36.2×	RBNY00000000	Dillon et al., 2019b
ICMP 13786*	5.99	58.0	5719	5066	606	42.8×	NZ_RBTN00000000	Dillon et al., 2019b

a Shading indicates genomes excluded from bioinformatics analyses because of the availability of genomes of higher quality for the same strain. PT, pathotype strain.

b ICMP 7711 and CFBP 5062; ICMP 16943 and CFBP 5067; CECT 4861 and ICMP 16945 correspond to the same P. savastanoi isolate obtained from two different collections (**Table 3**).

c NCPPP 3335 data include both the complete sequence of its chromosome (accession number NZ_CP008742.1) and those of its three native plasmids (pPsv48A, FR820585.2; pPsv48B, FR820586.1 and; pPsv48C, FR820587.2).

d National Centre for Biotechnology Information^1^.

Asterisks indicate oleander strains included in pathovar Psv (see **Figure 4**).

### DNA Manipulations

Basic DNA and molecular techniques followed standard methods (Sambrook and Russell, 2001). DNA sequences were visualized and manipulated using Geneious 8.1.9 (Kearse et al., 2012) and Artemis 16.0.0 (Carver et al., 2012).

Genomic DNA from Psv NCPPB 3335, Psn *Psn23*, Psr CECT 4861, and Psf NCPPB 1006 was extracted from cultures grown overnight using a Genomic DNA Purification JETFLEX kit (Genomed GmbH, Löhne, Germany), and purified by extraction with phenol-chloroform.

### Cross-Pathogenicity Tests

The pathogenicity of *P. savastanoi* strains was analyzed on *O. europaea* plants derived from a seed germinated *in vitro* (originally collected from an “Arbequina” plant), *N. oleander* plants accession “pink” (single pink flowers) supplied by Viveros Guzmán (Málaga, Spain), and *F. excelsior* and *R. sphaerocarpa* plants native from Valladolid and supplied by Viveros Fuenteamarga (Valladolid, Spain). For inoculation, bacterial cells grown on LB plates for 48 h at 28°C were collected, washed twice with 10 mM MgCl_2_ and adjusted to a final concentration of 10^8^ colony-forming unit (CFU)/ml in the same buffer. Olive, oleander, and ash plants were wounded with a scalpel along the stem and 10^6^ CFU were placed per wound (Penyalver et al., 2006; Pérez-Martínez et al., 2007). For broom plants, 10^7^ CFU were injected in each wound using a syringe with needle. For the cross-pathogenicity tests shown in **Figure 3** and **Supplementary Figure S3** we used two plants per strain. The number of wound sites infected per plant varied between 5 and 10, depending on the size of the plant. For the cross-pathogenicity tests of Psv and Psn strains shown in **Figure 4** we used three olive and three oleander plants per strain, with between 10 and 12 wounds sites infected per strain and plant. Wounds were protected with parafilm (Bemis, Neenah, WI, USA) for seven days to maintain humidity. Plants were kept in a greenhouse under natural photoperiod (15 h light/9 h dark) at room temperature (26°C day/18°C night). Symptoms were scored at 90 dpi and captured with a high-resolution digital camera (Nikon DXM 1200; Nikon Corporation, Tokyo, Japan), for olive, oleander, and ash, or with a stereoscopic microscope (Leica MZ FLIII; Leica Microsystems, Wetzlar, Germany), for broom plants. *P. savastanoi* cells were recovered from olive infected plants as follows. Three inoculated sites excised from two (**Figure 3**, **Supplementary Figure S3**) or three (**Figure 4**) different plants were homogenized by mechanical disruption, and serial dilutions plated on LB containing Nf and Ch; bacterial populations were estimated by colony counts.

### Bioinformatics Methods

#### Genome Sequencing and Assembly

Genome sequencing and assembly of the Psv NCPPB 3335 chromosome was performed at BGI Tech Solutions Co., Ltd. (Hong Kong). A library of randomly sheared DNA fragments (0.5-2.0 kb) was subjected to Illumina GA II (Solexa) sequencing. Reads (coverage, 200×) were qualitatively assessed before assembling with SOAP *de novo* (Li et al., 2008). Assembly of the NCPPB 3335 chromosome was aided by subtraction of the complete sequences of its three native plasmids (Bardaji et al., 2011). Primer walking and PCR amplification were used to fill the remaining gaps and solve misassembled regions. Sequencing of the Psn *Psn23*, Psr CECT 4861 and Psf NCPPB 1006 draft genomes was performed at Centro de Investigación Biomédica (CIBIR), La Rioja, Spain. Paired-end (100 cycles) sequencing used the Illumina GA ll platform (coverage, 250×). The genomes were assembled using CLC Genomics Workbench. The four genomes were automatically annotated upon submission to GenBank at National Centre for Biotechnology Information^1^ (NCBI). The accession numbers of these four genomes are listed in **Table 1**.

#### Core- and Pan-Genome Analyses

Core− and pan-genome analyses were performed using BPGA v1.3 (Chaudhari et al., 2016) with assemblies downloaded from the NCBI^1^. In all comparative analyses, Psv NCPPB 3335 was used as reference with the sequence of the complete chromosome and of its three plasmids concatenated in a single file (5′-chromosome-pPsv48A-pPsv48B-pPsv48C-3′). Orthologous genes were identified using the USEARCH algorithm (Edgar, 2010) with a threshold of 0.9 (90% blastp identity). To determine whether pan-genomes were opened or closed, we used the medians of the total number of genes found and the curves were then fitted to Heap’s law model (Tettelin et al., 2008).

The core- and pan-genome were also estimated using Roary v3.12.0 (Page et al., 2015) with ≥ 90% blastp identity and using the same assemblies as with BPGA, but annotated with Prokka (Seemann, 2014) using Galaxy v1.12.0^2^ (Afgan et al., 2016). The heat map depicting gene presence and absence was generated with Phandango (Hadfield et al., 2018).

#### Phylogenetic Analyses

Phylogenetic relationships were predicted by analysis of core genome single nucleotide polymorphisms (SNPs) using Parsnp v1.2 and Gingr v1.2 (Treangen et al., 2014), and 100 bootstrap replicates; trees were visualized and manipulated using MEGA 7 (Kumar et al., 2016). The *P. savastanoi* phylogeny was also analyzed by MLSA using 40 concatenated genes (**Supplementary Table S1**) whose sequences are complete in all 20 genomes (80,902-80,949 nt per strain). Sequence alignment using MUSCLE within MEGA 7 (Kumar et al., 2016) was hand curated to eliminate ambiguities. The tree was constructed using RaxML 7.2.8 within Geneious 8.1.9 (Kearse et al., 2012), with 200 bootstraps. All trees were rooted using *P. syringae* pv. ciccaronei ICMP 5710 (assembly LJPY01) as outgroup.

#### Prediction of Secretion Systems and Effectors

Codification of structural and regulatory components of the type III (T3SS), type IV (T4SS) and type VI (T6SS)secretion systems was investigated using T346 Hunter (Martínez-García et al., 2015a).

To search for known T3SS effectors (T3Es) we used PIFAR (Martínez-García et al., 2016). T3Es sequences were manually examined to search for truncations, disruptions, and frameshifts. To identify novel *hop* genes, we used and *ad-hoc* pipeline considering two different criteria: the N-terminal sequence features, using EffectiveDB^3^ (Eichinger et al., 2015), and presence of potential HrpL boxes 500 nucleotides upstream of the start codon, following previously described criteria (Fouts et al., 2002). Hierarchical clustering of *P. savastanoi* genomes based on their T3E content was performed using Morpheus^4^ with a presence-absence matrix with values of “1” (full-length CDS), “0.5” (truncated/disrupted/frameshifted CDS) and “0” (absence of T3E). Values of “2” and “1.5” were given for T3E paralogs encoded as two full-length CDSs or as one full-length and one truncated CDS, respectively. The matrix was used to measure the distance between strains using the Euclidean distance method. Then, the tree was constructed using the complete linkage hierarchical clustering method.

#### Identification of *P. savastanoi* Pathovar-Specific Genes and Virulence Factors

Variable regions (>3.9 kb) (**Supplementary Figure S1**) were identified with GView Pangenome Analysis tool^5^ (Petkau et al., 2010) using Psv NCPPB 3335 (5′-chromosome-pPsv48A-pPsv48B-pPsv48C-3′) as seed, and blastn with *e*-value ≤1 × 10^−10^ and ≥90% identity. IslandViewer4 (Bertelli et al., 2017) was used to predict genomic islands in the Psv NCPPB 3335 genome.

The gene content of *P. savastanoi* pathovars was compared using Roary v3.12.0 (Page et al., 2015) as described above. The resulting presence/absence matrix was used to obtain the lists of pathovar-exclusive genes and pathovar-absent genes. Genes were first classified by gene ontology (GO) enrichment analysis using Sma3s.v2 (Muñoz-Mérida et al., 2014) and then manually curated by blastx analysis (**Supplementary Table S2**).

Virulence gene repertoires were predicted using PIFAR (Martínez-García et al., 2016).

## Results

### General Genome Features

We reported the draft genome sequence of Psv NCPPB 3335 (Rodríguez-Palenzuela et al., 2010) and the complete sequence of its three plasmids (Bardaji et al., 2011). To use this genome as a reference in this and future comparative genomic analyses, we obtained the complete sequence of the Psv NCPPB 3335 chromosome. This yielded a single contig of 6.02 Mb, encoding 5,850 genes and 5,165 protein-coding sequences. Likewise, we generated draft genome sequences of three strains whose pathogenicity in their host of isolation was previously reported, *i.e.* Psn *Psn23*, Psf NCPPB 1006 and Psr CECT 4861 (**Table 1**). Additionally, we analyzed the previously published draft genomes of 19 other strains of the four *P. savastanoi* pathovars of woody hosts (**Table 1**). The relevant characteristics of all these genomes are shown in **Table 1**.

### The Pan-Genome and Core Genome

**Figure 1:**
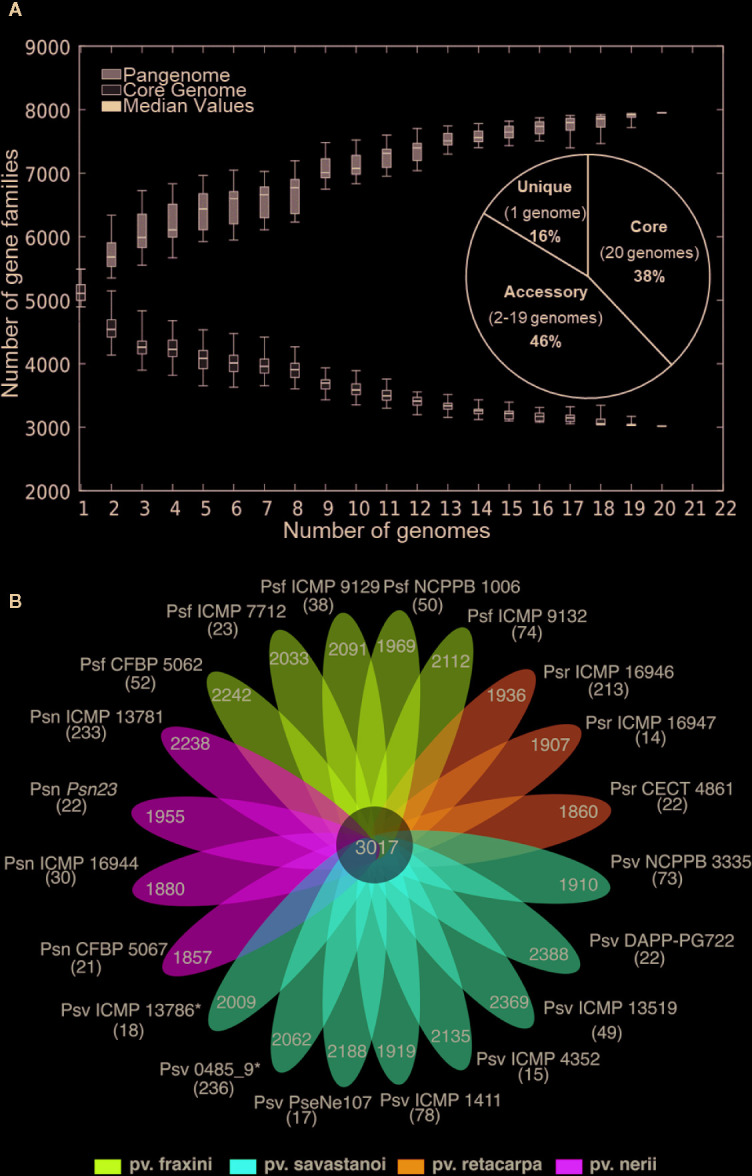
**(A)** Core- and pan-genome of *P. savastanoi* strains. Boxes indicate changes in number of gene families to the number of genes added sequentially, with median values denoted with a horizontal black line and standard deviation with vertical bars. The pie chart shows the frequency distribution of ortholog groups of genes. **(B)** Strain-specific *P. savastanoi* genes. Number of genes in the core-genome (center), accessory genes (petals) and strain-specific genes (in brackets), calculated using BPGA. Psv, Psn, Psf, and Psr, *P. savastanoi* pathovars savastanoi, nerii, fraxini, and retacarpa, respectively. Asterisks indicate oleander strains included in Psv (see **Figure 4**).

Six of the genomes correspond to three strains from different collections, independently sequenced by us and others (**Table 1**); we therefore selected those of the highest quality for subsequent genomic analyses. The resulting 20 genomes contained 114,601 genes, clustering into a hard pan-genome of 7,953 ortholog groups, of which 3,017 (38%) constitute the hard-core genome, 3,636 (46%) are accessory (encoded in 2-19 genomes), and the remaining 1,300 (16%) are strain-specific (**Table 2**, **Figure 1**). Nevertheless, there is a moderate strain-to-strain variation in the number of accessory genes (1,857 to 2,388) and strain-specific genes (14 to 236) (**Figure 1B**). We also estimated that only 19 additional ortholog groups are part of the *P. savastanoi* soft-core genome, *i.e.* 3,036 groups are present in at least 19 of the 20 genomes (95%).

**Table 2:** Comparison of the core genomes and pan-genomes of *Pseudomonas savastanoi* pathovars^a^.

Pathovar	Strains	Hard-core genome	Soft-core genome	Hard pan-genome	Soft pan-genome	Exclusive presence of genes	Exclusive absence of genes	Heap’s law (gamma)
All strains	20	3017	3036	7953	7937	–	–	0.1519
pv. fraxini	5	4652	4769	5738	5655	36	35	0.0652
pv. retacarpa	3	4282	4378	5846	5752	68	20	0.1395
pv. nerii	4	4107	4218	6201	6094	15	14	0.1431
pv. savastanoi	8	3804	3834	6766	6726	4	0	0.1200
**With original pv. assignation** ^b^								
pv. nerii	6	3689	3734	6720	6650	0	0	0.1512
pv. savastanoi	6	4309	4345	6166	6122	0	0	0.0937

a Hard, genes present in 100% of the genomes; soft, genes present in 95% of the genomes.

b Analyses of pv. nerii and pv. savastanoi considering the oleander strains 0485_9 and ICMP 13786 as belonging to pv. nerii, as they were originally classified. The other analyses were done including these two strains in pv. savastanoi, as indicated by the cross-pathogenicity tests.

**Figure 2:**
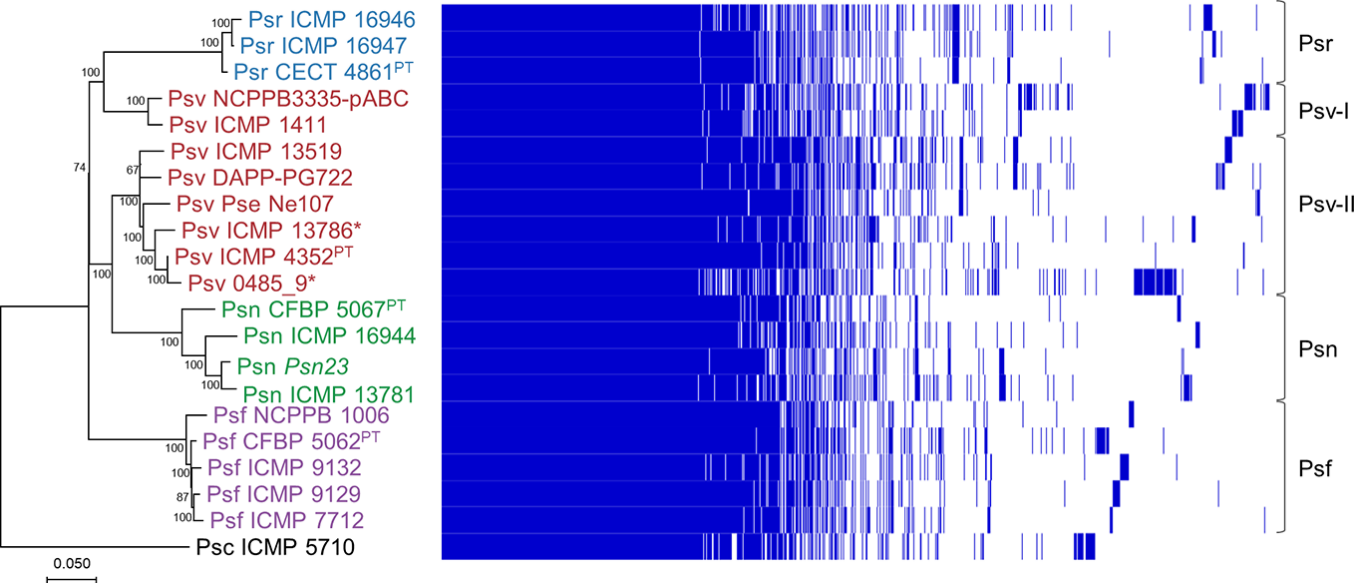
Phylogeny and gene content of *P. savastanoi* genomes. Maximum likelihood tree based on core genome SNPs, rooted with *P. syringae* pv. ciccaronei ICMP 5710. Values in nodes are bootstrap percentages from 100 replicates; the scale represents substitutions per site (left panel). Heat map representing presence (blue) or absence (blank) of individual genes (right panel). Strain abbreviations as in **Figure 1**; PT, pathotype strain.

Using the Heap’s law model, we estimated a fitting parameter (γ) of 0.15 for all 20 genomes (**Table 2**), just above the threshold of γ=0 distinguishing open (γ > 0) from closed (γ < 0) genomes. Therefore, sampling more *P. savastanoi* genomes will likely not result in a significant increase of the pan-genome size nor in a substantial reduction of the core genome, because their accumulation curves tend towards an asymptote (**Figure 1A**). A heat map representing presence and absence of individual genes on each strain is shown in **Figure 2**.

### Phylogeny of *P. savastanoi* Pathovars

We analyzed the phylogenetic relationships among the 20 P*. savastanoi* strains (**Table 1**) using SNPs from a core genome alignment. This tree (**Figure 2**) shows a very similar topology to a MLSA-based tree constructed using 40 concatenated genes (**Supplementary Table S1**, **Supplementary Figure S2**).

We could identify five well-differentiated clades (**Figure 2**), two of which correspond to pathovars Psr and Psf. However, pathovar Psv appears not to be monophyletic being distributed into two different clades. Two strains of Psv (NCPPB 3335-pABC and ICMP 1411) clustered closely to the Psr strains (Psv-I cluster), well-separated from the remaining Psv strains (Psv-II cluster). This last group also includes two oleander strains (ICMP 13786 and 0485_9), whereas the remaining Psn strains clustered together in a separate clade.

### Host Range of *P. savastanoi* From Woody Hosts

**Figure 3:**
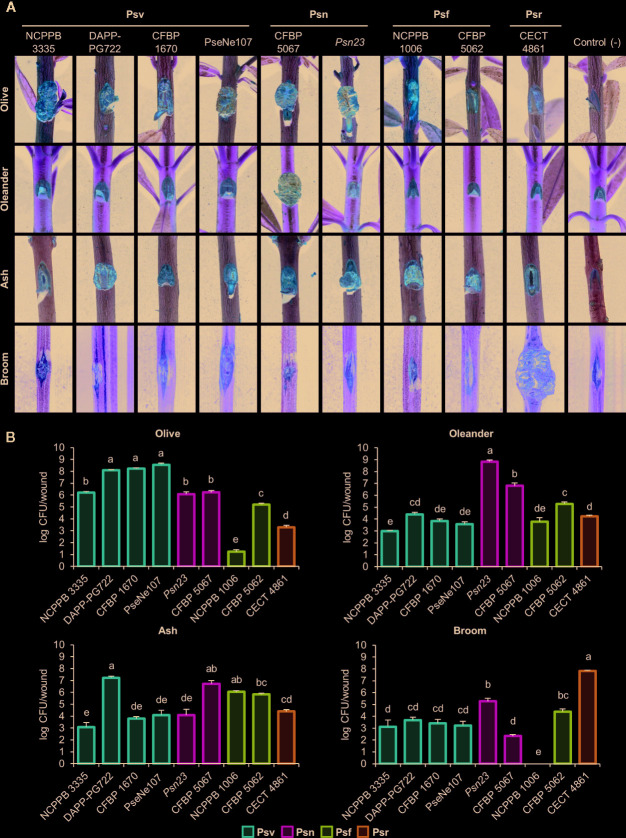
Cross pathogenicity tests of *P. savastanoi* strains. **(A)** Symptoms generated by *P. savastanoi* strains at 90 days post-inoculation. Control (-), negative control, plants inoculated with 10 mM MgCl_2_. **(B)** Populations of *P. savastanoi* strains in different hosts. Bars represent mean populations at 90 days post-inoculation from three biological replicates, with standard error; different letters indicate means that are significantly different using ANOVA test followed by the Bonferroni t-test (p < 0.05). Psv ICMP 4352 was obtained from the CFBP collection as strain CFBP 1670. Psv, Psn, Psf and Psr, *P. savastanoi* pathovars savastanoi, nerii, fraxini, and retacarpa, respectively.

The pathogenicity of the nine *P. savastanoi* strains whose genomes were available in 2017 (**Figure 3**, **Table 3**, **Supplementary Figure S3**) was compared with published host ranges (Janse, 1991; Álvarez et al., 1998; Iacobellis et al., 1998).

**Table 3:** Wild-type *Pseudomonas savastanoi* strains used in cross-pathogenicity tests.

Pathovar	Host of isolation	Strains^a^					Country of isolation	Isolation year	Reference/Original source
		CECT	CFBP	ICMP	NCPPB	Other			
**pv. fraxini**	*Fraxinus excelsior*		1663	4347	**1006**		United Kingdom	1961	Janse (1981)
			**5062** ^b^	7711			Netherlands	1978	Janse (1991)
**pv. nerii**	*Nerium oleander*					***Psn23*/ESC23**	Italy	2004	Tegli et al. (2011)
			**5067** ^b^	16943	3278		Spain	2007	Janse (1991)
				**13781**		ITM310	Italy	Before 1985	Sisto et al. (2007)/N.S. Iacobellis
				**16944**	3334		France	1984	D.E. Stead
**pv. retacarpa**	*Retama sphaerocarpa*	**4861** ^b^	5512	16945	4050		Spain	1996	Álvarez et al. (1998)
**pv. savastanoi**	*Olea europaea*	5024			**3335**		France	1984	Pérez-Martínez et al. (2007)/D.E. Stead
			**1670** ^b^	4352	639		Serbia	1959^c^	D. Sutic
						**DAPP-PG722**	Italy	2007	Hosni et al. (2011)
						**PseNe107**	Nepal	2007	Balestra et al. (2009)
				**13519**			New Zealand	1997	J.M. Young
		5021		**1411**	1344^d^	ICPB PS170	USA	1961	E.E. Wilson
	*Nerium oleander*					**0485_9**	USA	1985	Azad & Cooksey (1995)
				**13786**		ITM601	Italy	1985	Morea et al., 1989/N.S. Iacobellis

a Strains used in this study for experimental analyses are highlighted in bold. CECT, Colección Española de Cultivos Tipo; CFBP, Collection Française de Bactéries Phytopathogènes; ICMP, International Collection of Microorganisms from Plants; NCPPB, National Collection of Plant Pathogenic Bacteria; ITM, Istituto Tossine e Micotossine da Parassiti Vegetali Collection (Bari, Italy); DAPP, Dipartimento di Scienze Agrarie, Alimentari e Ambientali, Università degli Studi di Perugia (Perugia, Italy); ICPB, International Collection of Phytopathogenic Bacteria.

b Pathotype isolates; P. savastanoi pv. savastanoi CFBP 1670 is considered both P. savastanoi type strain and P. savastanoi pv. savastanoi pathotype strain.

c Year of addition to NCPPB.

Psr CECT 4861 induced well-defined knots at most inoculation sites only on broom plants (**Figure 3A**, **Supplementary Figure S3**). Ash and olive plants showed tissue swelling at most inoculation sites (80% and 60%, respectively), while infected oleander plants remained symptomless (**Figure 3A**, **Supplementary Figure S3**). These results agree with the narrow host range reported for *P. savastanoi* isolates from broom (Álvarez et al., 1998).

On ash plants, Psf strains NCPPB 1006 and CFBP 5062 induced excrescences (necrotic swellings) at most inoculation points (**Figure 3A**, **Supplementary Figure S3**). Excrescences also appeared in up to a third of infected sites on olive plants, supporting the reported ability of Psf to also induce symptoms in this host (Janse, 1981; Janse, 1982; Iacobellis et al., 1998). Inoculated oleander plants were symptomless.

All four Psv strains induced visible symptoms on olive and ash plants, although they were variable and strain dependent (**Figure 3A**, **Supplementary Figure S3**). For instance, Psv NCPPB 3335 and Psv PseNe107 generated knots on olive plants, which were more necrotic on wounds inoculated with the later, while Psv CFBP 1670 and Psv DAPP-PG722 primarily induced swelling of the tissues surrounding the wounds. This agrees with the reported strain- and cultivar-dependent severity of symptoms in inoculated olive plants (Penyalver et al., 2006; Pérez-Martínez et al., 2007). Ash plants primarily developed either knots (Psv DAPP-PG722) or swellings (remaining strains). Conversely, most Psv-infected oleander and broom plants remained symptomless and only a reduced number of wounds showed slightly swollen lesions (**Figure 3A**, **Supplementary Figure S3**).

Psn *Psn23* and CFBP 5067 induced necrotic knots on olive plants and small overgrowths on ash. Most oleander plants inoculated with Psn *Psn23* developed knots, however, few showed symptoms with Psn CFBP 5067 (**Figure 3A**, **Supplementary Figure S3**), perhaps reflecting the inability of some Psn isolates to induce knots in certain oleander cultivars (Janse, 1981). Nevertheless, both Psn CFBP 5067 and *Psn23* appear to induce systemic infections because inoculated oleanders showed secondary symptoms, such as flower bud swellings, leaf curling, and tiny knots on the leaves (see **Supplementary Figure S4** for CFBP 5067). Broom plants infected with Psn showed no symptoms (**Figure 3A**, **Supplementary Figure S3**).

All the strains reached the highest populations densities at 90 days post-inoculation (dpi) in their host of isolation, with medium to low populations in the other hosts. No viable counts were recovered from symptomless broom plants infected with Psf NCPPB 1006 (**Figure 3B**).

In summary, our results agree with the host range previously reported for the four well-established *P. savastanoi* pathovars of woody hosts. Additionally, we also found that some Psv strains can occasionally induce the formation of slightly swollen lesions on broom, and that strain Psr CECT 4861 also induces the formation of slight symptoms at some inoculation sites not only on broom, but also on olive and ash (**Figure 3A**, **Supplementary Figure S3**).

### Phylogeny of *P. savastanoi* Strains Isolated From Olive and Oleander Reflects Differential Pathogenicity in These Two Hosts

Psv strains did not cluster in a monophyletic lineage and two oleander strains clustered with four olive strains (**Figure 2**, **Supplementary Figure S2**). To confirm their pathovar assignation, we therefore examined the pathogenicity in olive and oleander of the six olive and oleander strains not included in the cross-pathogenicity tests shown in **Figure 3**.

**Figure 4:**
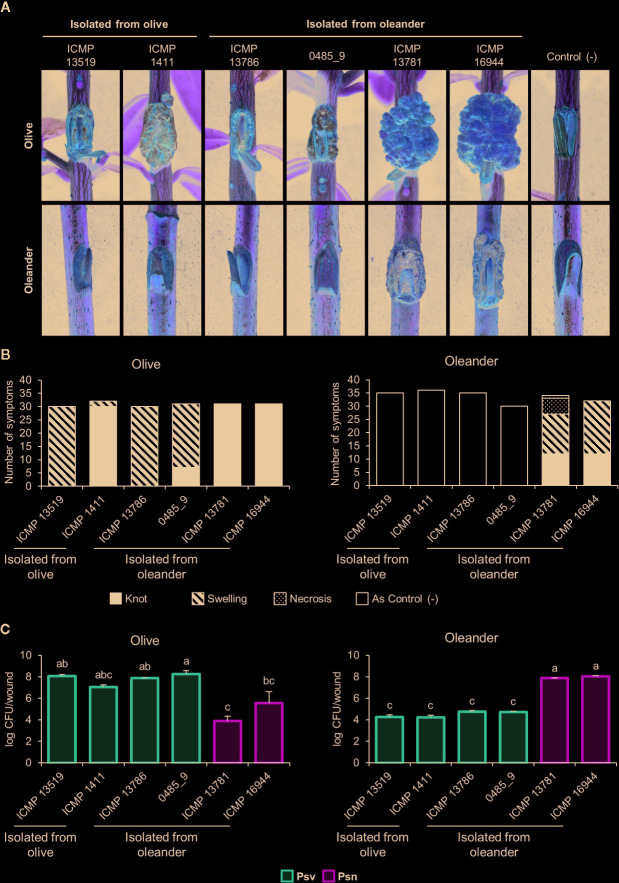
Cross pathogenicity tests of *P. savastanoi* pv. savastanoi and nerii strains. **(A)** Symptoms at 90 days post-inoculation. C(-), negative control, plants inoculated with 10 mM MgCl_2_. **(B)** Number of inoculation points showing differential symptoms. **(C)** Populations of *P. savastanoi* pv. savastanoi (Psv) and pv. nerii (Psn) strains at 90 days post-inoculation. Details as in **Figure 3**.

Psn strains ICMP 16944 and ICMP 13781 induced the largest knots in olive plants, but their bacterial populations were the lowest (**Figure 4**). Additionally, they were the only strains inducing knots or swellings on oleander plants, were they reached very high populations. Conversely, oleander strains ICMP 13786 and 0485_9 induced symptoms in olive plants, were they reached the highest bacterial populations, but not in oleander. Thus, these two strains resemble the typical behavior of Psv strains and, although isolated from oleander, they belong to pathovar savastanoi.

### Secretion Systems

The distribution of the T3SS, T4SS, and T6SS determinants was investigated using the web-based tool T346Hunter (Martínez-García et al., 2015a).

**Figure 5:**
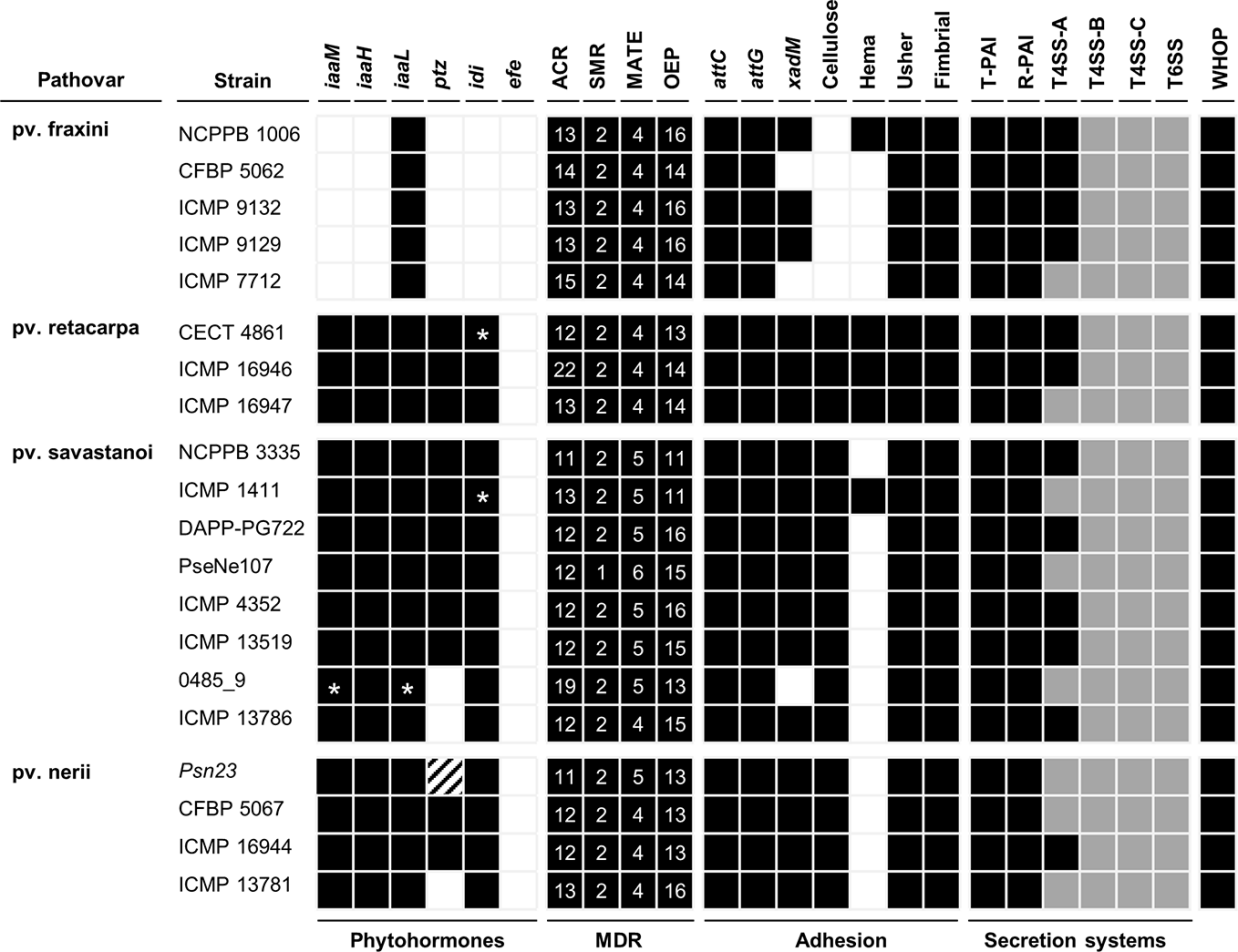
Bioinformatics prediction of the virulence gene repertoires of *P. savastanoi* strains using PIFAR. Black boxes and white boxes, presence or absence, respectively, of the indicated gene or gene set; asterisks, genes not found in the genome but identified by PCR; grey boxes, partial codification of core genes; striped boxes, genes not found in the assembly but found in the unassembled reads. MDR, multidrug resistance transporter; ACR, acridine-like transporter from the resistance/nodulation/cell division (RND) family; SMR, small multidrug transporter family; MATE, multidrug and toxic compound extrusion family; OEP, outer membrane efflux protein (RND family); *attC* and *attG*, attachment gene homologs (*Agrobacterium tumefaciens*); *xadM*, adhesion gene homolog (*Xanthomonas oryzae*); Cellulose, cellulose synthase; Hema, hemagglutinin-repeat protein; Usher, outer membrane usher protein; fimbrial, fimbrial protein. T-PAI, canonical tripartite T3SS; R-PAI, rhizobial T3SS; T4SS, type IV secretion system; T6SS, type VI secretion system. WHOP, genomic island carrying four operons and other genes involved in degradation of phenolics (Caballo-Ponce et al., 2017b).

As in Psv NCPPB 3335 (Rodríguez-Palenzuela et al., 2010), all the genomes encoded two different T3SS clusters: the tripartite pathogenicity island, the most prevalent T3SS within the *P. syringae* complex (Baltrus et al., 2017; Dillon et al., 2019b), and an additional T3SS resembling that found in *Rhizobium* species (**Figure 5**) (Joardar et al., 2005; Martínez-García et al., 2015b). Although most of the corresponding deduced products showed 100% identity among genomes, we also found amino acid variations characteristic of each of the five phylogenetic groups (**Table 4**).

**Table 4:** Amino acids replacements in structural and regulatory proteins of the type III secretion system in *P. savastanoi* pathovars of woody hosts.

Protein	Pathovar/Phylogenetic group/strain^a^	No. of replacements	Position in the protein	Amino acid in Psv NCPPB 3335^b^	Amino acid replacement	Type of replacement^c^
HrpL	Psr	1	63	His (H)	Gln (Q)	R
	Psf	2	38	Gln (Q)	Arg (R)	C
			106	Gly (G)	Glu (E)	R
HrpQ	All *P. savastanoi* (except for Psv-I)	1	195	His (H)	Arg (R)	R
HrcR	Psn	1	24	Leu (L)	Met (M)	C
HrpV	Psv-II and Psn	1	73	Phe (F)	Val (V)	R
HrcC	Psv-II and Psn CFBP 5067	1	273	Gly (G)	Val (V)	R
	Psf	1	579	Val (V)	Leu (L)	C
	Psn (except for Psn CFBP 5067)	2	273	Gly (G)	Val (V)	R
			699	Lys (K)	Gln (Q)	C
HrpD	Psr	1	145	Ala (A)	Val (V)	R
	Psn ICMP 16944	1	123	Pro (P)	Thr (T)	R
HrcJ	Psf	1	80	Glu (E)	His (H)	C
HrpZ	Psv-II	1	4	Leu (L)	Phe (F)	R
HrpR	Psf	1	2	Ser (S)	Asn (N)	C

a Psv, Psn, Psf, and Psr, P. savastanoi pathovars savastanoi, nerii, fraxini, and retacarpa, respectively. Phylogenetic groups from **Figure 2**: Psv-I (Psv NCPPB 3335 and Psv IMCP 1411), Psv-II (Psv ICMP 4352, Psv PseNe107, Psv DAPP-PG722, Psv ICMP 13519, Psv 0485_9, and Psv ICMP 13786), Psn (Psn ICMP 13781, Psn Psn23 and Psn CFBP 5067).

b Psv NCPPB 3335 was used as reference genome.

c C and R, replacements to an amino acid with similar or different biochemical properties than the original, respectively.

The complete set of T4SS-A genes (*virB1-virB11* and *virD4*) carried by plasmid pPsv48B (Bardaji et al., 2011) was found in 12 genomes (**Figure 5**). Additionally, all 20 strains carried incomplete sets of T4SS-B genes (*tra* and/or *trb* genes) and T4SS-C genes (*tfc* genes) (**Figure 5**). The T4SS-C is involved in propagation of genomic islands (Juhas, 2015), and has not been previously described in the *P. syringae* complex. As described for Psv NCPPB 3335 (Rodríguez-Palenzuela et al., 2010), *P. savastanoi* strains encode two different T6SS clusters, one highly similar to that found in *P. syringae* pv. phaseolicola 1448A and a second homologous to the T6SS *hcp1* cluster of *P. syringae* pv. tomato DC3000. Considering the composition of both T6SS clusters and additional chromosomal genes, all 20 genomes contains 12 of the 13 genes constituting the T6SS core (Boyer et al., 2009), only lacking that encoding the forkhead-associated (FHA) domain-containing protein VCA0112 (**Figure 5**).

### Distribution of T3SS Effectors

**Figure 6:**
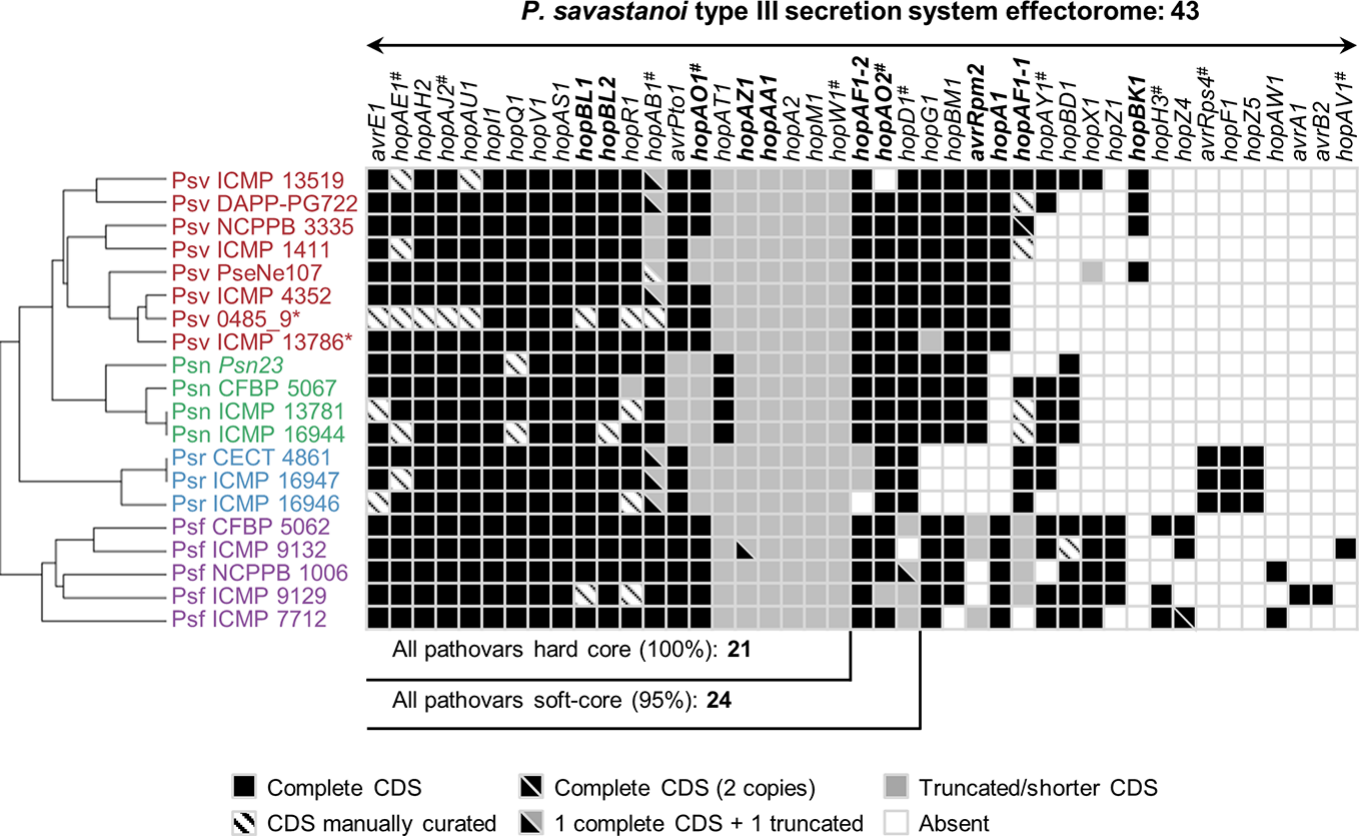
Distribution and hierarchical clustering of known type III secretion system effector (T3E) families. Bolded names, T3Es whose translocation has been previously demonstrated in *P. savastanoi* (Caballo-Ponce et al., 2017a). Hashes, T3E family names merged into new families (HopAE/HopW=HopW, HopAB/HopAY=HopAB, HopAO/HopD=HopD, HopK/AvrRps4=HopK) and T3E alleles established as novel families (HopAJ2=HopBV, HopH3=HopBQ, and HopAV1=HopBS) (Dillon et al., 2019a). Strain abbreviations as in **Figure 1**; CDS, coding sequence. The hierarchical clustering (left) was generated using Morpheus.

**Figure 7:**
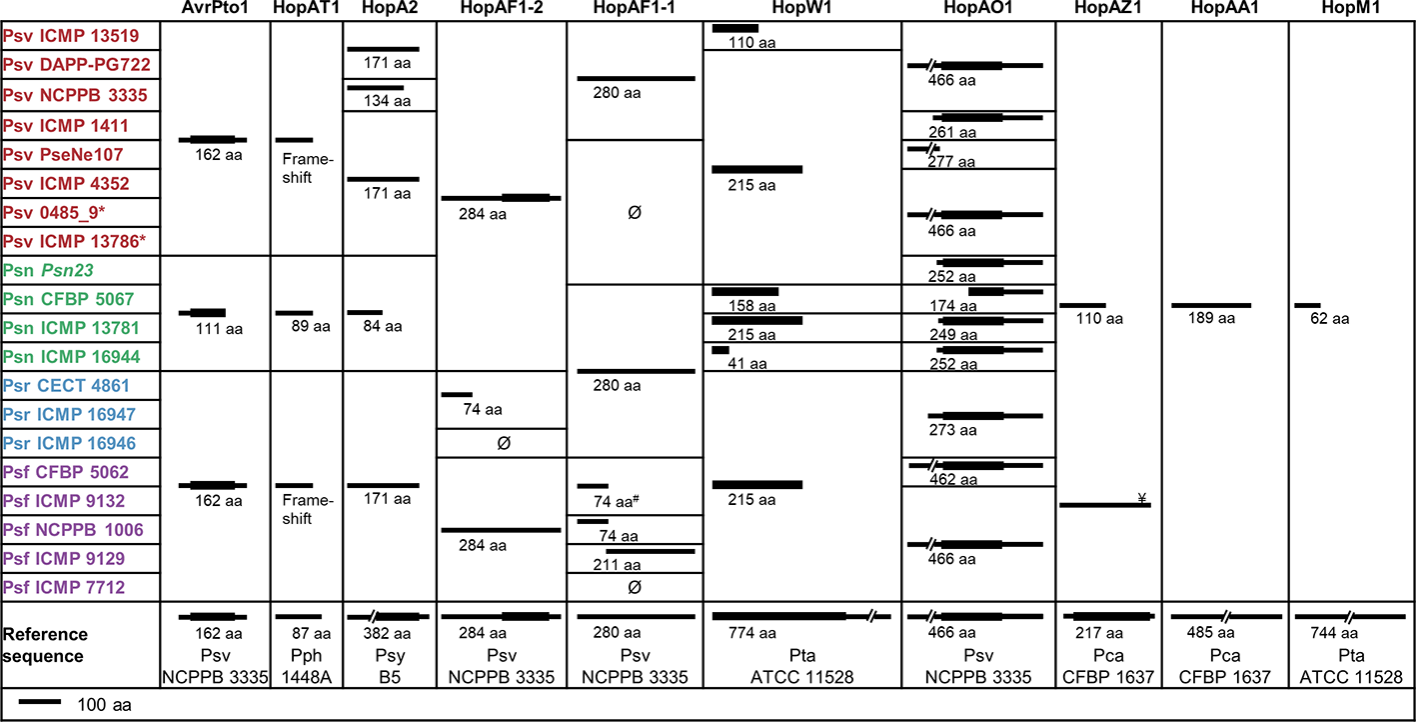
Truncated T3Es in *P. savastanoi* strains. Black lines represent the sequence length of the deduced T3E product and black boxes represent Pfam^7^ or HHPred^8^ protein domains. ^Ø^ and ^#^ indicate the absence and the truncation of *hopAF1-1* by insertion of an IS*5*-like element, respectively. ^¥^Psf ICMP 9132 encodes a full-length *hopAZ1*; however, the encoded protein does not contain the functional domain found in the reference sequence. Strain abbreviations as in **Figure 1**. Pca, Pph, Psy, and Pta, *P. cannabina* pv. alisalensis, *P. syringae* pv. phaseolicola*, P. syringae* pv. syringae, and *P. syringae* pv. tabaci, respectively. ^7^https://pfam.xfam.org/ ^8^https://toolkit.tuebingen.mpg.de/tools/hhpred

The *P. savastanoi* pan-genome encodes 43 known T3Es (**Figure 6**), including truncated genes, distributed in 37 T3E families from the Hop database^6^ and 33 of the new merged families (Dillon et al., 2019a). T3E truncations were analyzed and assigned to groups according to their extent (**Figure 7**). All the strains contained the same truncations of *hopAZ1*, *hopAA1,* and *hopM1*. Conversely, T3E allelic variants encoded by specific pathovars, and therefore possibly involved in host specificity, include those only found in all Psn (*avrPto1*, *hopAT1,* and *hopA2*) or Psr strains (*hopAF1-2* and *hopAO1*). Additionally, while all Psv and Psn strains encode a full-length coding sequence (CDS) for *avrRpm2*, Psr, and Psf strains either encode a truncated version of this gene or do not contain it. Specific truncations affecting single strains were also identified for *hopA2*, *hopAF1-1*, *hopAO1,* and *hopW1* (**Figure 7**).

We found two novel T3E candidates not homologous to known T3Es (**Table 5**) but preceded by a *hrp* box promoter, characteristic of genes regulated by HrpL (Fouts et al., 2002). Additionally, their deduced products meet at least two of the three features of N-terminal secretion signals found in Hop proteins (Lindeberg et al., 2005). The gene coding for WP_032074452 was found in all 20 P*. savastanoi* genomes, whereas that for WP_057446723 was exclusively found in Psr genomes.

**Table 5:** Novel putative type III effectors identified in *Pseudomonas savastanoi*.

						T3SS signal in the N-terminus^d^			**Orhology**			
Accession number^a^	Protein ID	Annotation	Pfam domain^b^	Size (aa)	*hrp* value^c^	A	B	C	Psv	Psn	Psf	Psr
PSA3335_RS11510	WP_032074452	DUF72 domain-containing protein	PF01904	289	2577	+	+	+	+	+	+	+
B7R56_RS15315	WP_057446723	Hypothetical protein	Not found	304	2558	+	−	+	−	−	−	+

a PSA3335_RS11510 and B7R56_RS15315 correspond to the Psv NCPPB 3335 and Psr CECT 4861 genomes, respectively.

b The Pfam database (https://pfam.xfam.org/).

c hrp value, counter variable used to identify *hrp* boxes in the promoter regions, 500 nucleotides before the start of the gene.

d Prediction of N-terminal secretion signal in Hop proteins. A, high percentage of serine in the 50 N-terminal amino acids; B, isoleucine, leucine, valine, alanine, or proline in the third or fourth N-terminal amino acids; C, absence of acidic residues within the 12 N-terminal amino acids (Lindeberg et al., 2005).

Asterisks indicate oleander strains included in pathovar Psv (see **Figure 4**).

e Psv, Psn, Psf and Psr, P. savastanoi pathovars savastanoi, nerii, fraxini and retacarpa, respectively; + = presence, - = absence of a protein ortholog in the genomes of all strains included in the corresponding pathovar analyzed in this study **Table 1**.

Hierarchical clustering based on T3E content identified three main clusters composed of i) all Psf genomes (Psf cluster), ii) all Psr genomes (Psr cluster) and iii) all Psv and Psn strains (Psv-Psn cluster) (**Figure 6**). The Psv-Psn cluster was divided into two sub-clusters, one comprising all eight Psv strains and the other the four Psn strains (**Figure 6**). Thus, T3E clustering of *P. savastanoi* genomes is similar to the core genome phylogeny (**Figure 2**) indicating that the four pathovars contain characteristic sets of T3Es, which are thus likely contributing to define their pathogenicity profile.

We found 21 core *P. savastanoi* T3E genes, encoded as full-length ORFs or truncated versions in all the genomes. Three additional T3Es (*hopAF1*-2, *hopAO2,* and *hopD1*) were found in 19 of the 20 genomes analyzed (95%) and were included in the soft-core effectorome. Four T3E genes are candidates to contribute to host range definition: the Psr-exclusive *avrRps4*, *hopF1,* and *hopZ5* genes, and the *hopG1* gene, only absent in all three Psr strains (**Figure 6**). Known T3E encoded in single pathovars but not in all strains, thus likely not having a relevant role in host specificity, are: *hopBK1* (Psv strains), *hopZ1*, *hopH3*, *hopZ4*, *hopAW1*, *avrA1*, *avrB2,* and *hopAV1* (Psf strains).

### Identification of Pathovar-Specific Genes

**Figure 8:**
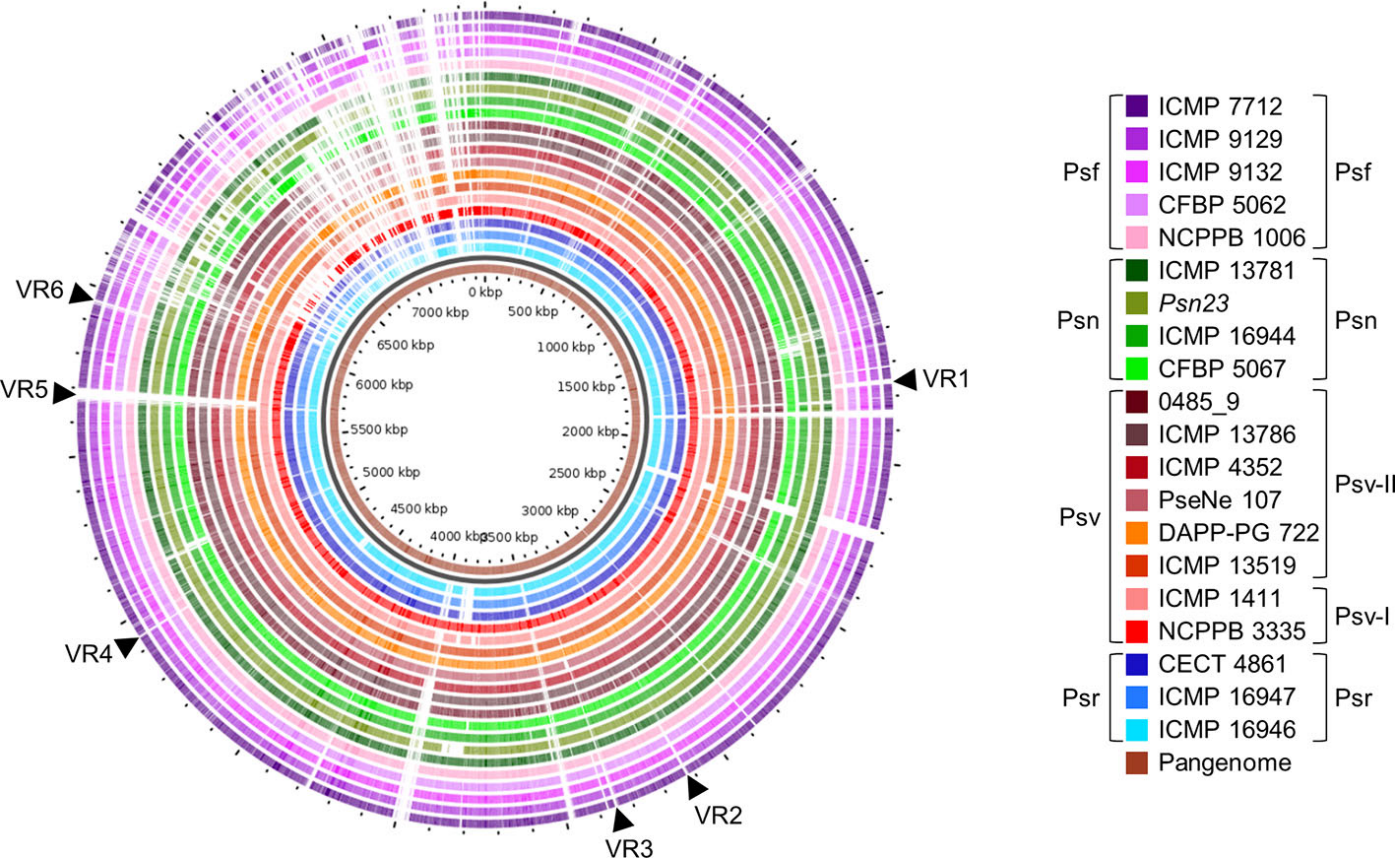
Genomic comparison of *Pseudomonas savastanoi* pathovars. Comparison made using the GView Pangenome analysis tool. Black arrows, selected variable regions (VR) that could be pathovar-specific (see **Supplementary Figure S1**). Strain abbreviations as in **Figure 1**, indicating the phylogenetic grouping from **Figure 2** to the right of the legend.

We identified several variable regions (VR) longer than 3.9 kb that were exclusively present or absent in distinct monophyletic groups (**Figure 8**). In general, the patterns of presence/absence of these VRs are congruent with the organismal phylogeny (**Figure 2**) and suggest their acquisition before pathovar differentiation followed by further vertical inheritance. Six VRs were of interest for being chromosomal and exclusively absent from all Psf strains (**Figure 8**, **Supplementary Figure S1**). VR3, VR4, VR5, and VR6 are enriched in hypothetical proteins (HPs) whereas VR1 and VR2 contain genes encoding putative virulence factors. VR1 encodes the *iaaMH* operon, responsible for the biosynthesis of indole-3-acetic acid (IAA) *via* indole-3-acetamide, and two genes involved in rhizobitoxine biosynthesis (*rtxA* and *rtxC*), a phytotoxin modulating plant-microbe interactions by inhibition of ethylene synthesis (Sugawara et al., 2006). In turn, VR2 encodes the type II secretion system proteins GspH and GspI and a pectate lyase (**Figure 8**, **Supplementary Figure S1**). With the exception of VR2, the other five VRs were predicted as genomic islands, being also often bordered by mobile genetic elements.

The core and pan-genome profiles of the 20 strains were also analyzed at the pathovar level using BPGA (**Table 2**). Psf showed the largest pathovar soft-core genome size, with a nearly closed pan-genome (γ=0.065), suggesting that the gene content of Psf strains is more homogeneous than that of the other three pathovars. We also conducted this analysis considering the original assignation of strains 0485_9 and ICMP 13786 to pv. nerii (**Table 2**), which completely prevented the identification of exclusively present/absent genes in both Psn and Psv. This illustrates the confounding effects that might originate from an improper identification of the strains under analysis, and reinforces the need for appropriate phenotypic analyses to support conclusions derived exclusively from genomic data.

We explored the gene repertoire specific to pathovars using two complementary bioinformatics tools, Roary (Page et al., 2015) (**Supplementary Table S2**) and PIFAR (**Figure 5**) (Martínez-García et al., 2016). A total of 123 pathovar-exclusive genes and 69 pathovar-absent genes were identified and classified into eight categories, among which HPs and metabolism-related genes were the most common (**Table 2**, **Supplementary Table S2**). Many of the pathovar-exclusive and pathovar-absent genes are likely carried by plasmids and other mobile genetic elements (**Figure 8**, **Supplementary Figure S1**), because we found a fair number of genes characteristic of these elements, such as genes involved in DNA replication, recombination, mutation, and repair, and toxin/antitoxin systems (Bardaji et al., 2019). Although around 24% of the pathovar-exclusive/absent genes encoded HPs, other pathovar-exclusive genes encode proteins with a possible role in virulence and/or host specificity, such as 11 transcription factors, three c-di-GMP-related proteins, two chemotaxis-related proteins, 18 transporters belonging to eight superfamilies, the Psn-exclusive secretion systems chaperones PapD (type I secretion system; T1SS) and ShcF (T3SS), four Psr-exclusive T3Es (see also **Figure 6** and **Table 5**) and the ferric-pseudobactin receptor PupB, also exclusive of Psr strains.

In addition to the absence in all Psf genomes of the *iaaMH* operon and the rhizobitoxin genes (**Figure 5**, **Supplementary Table S2**), other Psf-absent genes include those required for cytokinins (*ptz*) and cellulose biosynthesis, the type II secretion system (T2SS) genes *hxcU*, *hxcP,* and *hxcV*, the bacteriocin-related gene *ALO43_200111*, two genes involved in c-di-GMP turnover (*gmr_3* and *gmr_8*), three genes encoding transporters of the ATP-binding cassette (ABC) and LysE superfamilies and *cph2*, encoding a bacteriophytochrome (**Figure 5**, **Supplementary Table S2**). All these genes are shared by all three tumorigenic *P. savastanoi* pathovars and could therefore be essential for knot induction on diverse plant hosts. Among others, Psn strains lack genes encoding an enzyme required for the biosynthesis of the phytotoxin phevamine A (O’Neill et al., 2018).

## Discussion

Genomics-based methods offer unprecedented resolution to analyze strain diversity and evolutionary relationships within the *P. syringae* complex, besides identifying candidate genes involved in pathogenicity and host range definition (Lindeberg et al., 2009; Baltrus et al., 2011; Bartoli et al., 2015; Monteil et al., 2016; Baltrus et al., 2017; Dillon et al., 2019a; Dillon et al., 2019b). However, understanding the biological meaning of the observed genomic differences depends on the availability of experimental data confirming specific phenotypes, such as virulence and host range (Baltrus and Orth, 2018). Specifically, testing more than one hundred *P. syringae* strains on different hosts showed that the host range is an overlapping continuum, with closely related strains exhibiting from extremely narrow to very broad host ranges (Morris et al., 2019). Our genomic analyses of 20 strains representing all four well-established *P. savastanoi* pathovars of woody hosts were combined with cross-pathogenicity tests, supporting the identification of candidate genes involved in host range definition. In fact, our combination of bioinformatics and functional analyses was critical to guarantee a correct pathovar assignation, ensuring the correct interpretation of the comparative data.

### The *P. savastanoi* Pathovars Pan-Genome

The *P. savastanoi* pan-genome (7,953 ortholog families for 20 genomes) (**Table 2**) is approximately 10 and 3.5 times smaller than those of the *P. syringae* complex (391 genomes) and PG3 (143 genomes), respectively (Dillon et al., 2019b). Additionally, the pan-genomes of the *P. syringae* complex and PG3 are still open (Dillon et al., 2019b), whereas the pan-genome for each *P. savastanoi* pathovar is nearly closed (**Table 2**). The large homogeneity among *P. savastanoi* genomes results from a low content of pathovar- and strain-specific genes (16% strain-specific genes vs. ca. 60% in the *P. syringae* complex; **Figure 1**), probably reflecting close phylogenetic relationships, a large repertoire of shared niche-specific genes and, perhaps, a natural inability to acquire foreign DNA (Pérez-Martínez et al., 2007). However, it is possible that the analyzed genomes may not represent the true diversity of the species and its four pathovars of woody hosts. Thus, the analysis of additional *P. savastanoi* genomes from strains isolated from woody host, as well as from the surface of herbaceous plant hosts or from non-agricultural environments, could contribute to the genetic diversity of this pathogen, resulting in more open pangenomes and reducing the number of host range determinants identified in this study. In fact, other authors have reported the existence of further genotypical and phenotypical variability among *P. savastanoi* strains, such as the ability to produce levan or an atypical host range (Marchi et al., 2005; Sisto et al., 2007; Cinelli et al., 2014; Moretti et al., 2017; Morris et al., 2019).

### Correlation Between Phylogeny and Pathogenicity

Seven of the strains analyzed here were repeatedly shown to cause distinct syndromes in their host of isolation (Psv NCPPB 3335, DAPP-PG722, and ICMP 1411; Psn *Psn23*; Psf NCPPB 1006 and CFBP 5062; Psr CECT 4861). However, pathogenicity data were not available for the remaining 13 strains, which were assigned to a *P. savastanoi* pathovar (**Tables 1**, **3**) based on their host of isolation and, in some cases, on biochemical tests.

The cross-pathogenicity tests for nine *P. savastanoi* strains in olive, oleander, ash, and broom plants (**Figure 3**, **Supplementary Figure S3**) agree with the host range reported for the four well-established *P. savastanoi* pathovars of woody hosts. This supports using their genomes for comparative genomics analysis to identify candidate genes involved in host range definition. Psv, Psn, and Psf strains showed differences in virulence and in the ability to grow and survive in plant tissues, agreeing with previously reported cultivar-dependent virulence variability (Penyalver et al., 2000; Moretti et al., 2017). Additionally, we show that symptom severity is not always correlated with population levels *in planta*. In general, *P. savastanoi* strains showed the highest population level in their host of isolation (**Figure 3B**), regardless of their ability to also induce visible symptoms on other hosts. Nevertheless, maintenance of reduced bacterial density in non-host plants could favor the appearance of better-adapted variants and the development of emerging diseases in new hosts.

Agreeing with previous data (Gomila et al., 2017; Dillon et al., 2019b), Psf, Psn, Psr, and Psv cluster in a discrete genomic branch in the SNP ML tree (**Figure 2**) with non-tumorigenic pathovars as the phylogenetically closest relatives (Dillon et al., 2019b). This suggests that Psf, Psn, Psr, and Psv originate from a common ancestor, which likely acquired the ability to induce tumors in plants. These four pathovars also corresponded with well-defined genetic lineages indicating distinct evolutionary events leading to host specialization, although it is necessary to make two considerations. First, the Psv genomes are distributed in two separated clades, clustering with either Psr or Psn genomes (**Figure 2**). Although intriguing, the occurrence of different genetic lineages for a given pathovar is not new nor surprising (Gironde and Manceau, 2012; Martín-Sanz et al., 2012; Martín-Sanz et al., 2013; Hulin et al., 2018), because pathovars are defined for causing distinctive disease syndromes and not necessarily for their genetic relatedness (Young et al., 1992). Nevertheless, olive is a frequent host for the four pathovars and, since strains of *P. savastanoi* can cause knots in diverse species (Caballo-Ponce et al., 2017a), it is also possible that the two Psv genetic lineages show differing host ranges. In fact, pathogenicity assays on 15 herbaceous and woody hosts showed that the host range of Psv PseNe107 (Psv-II) is broader than that of Psv NCPPB 3335 (Psv-I) (Morris et al., 2019). A second relevant consideration is the clustering with Psv strains of two putative Psn strains (**Figure 2**). These two strains were more like Psv than Psn for several characteristics, such as their virulence gene repertoire (**Figure 5**) or their pattern of T3E genes (**Figure 6**). Nevertheless, the ability of strains 0485_9Ĕ and ICMP 13786 to cause knots in olive but not in oleander, and their better survival in olive than in oleander tissues (**Figure 4**) warrants their unequivocal classification as Psv strains (Janse, 1982).

### T3SS and Effectors

All 20 genomes encode a canonical T-PAI T3SS cluster (**Figure 5**, **Table 4**). Since it is essential for knot formation and the induction of a visible hypersensitive response (HR) by Psv and Psn (Caballo-Ponce et al., 2017a), and given its high sequence and functional conservation (**Table 4**) (Büttner and He, 2009), we could expect a similar role for Psf and Psr. The number of T3Es in *P. syringae* complex strains varies from four to nearly 50 (Dillon et al., 2019a), and the average in woody hosts strains is 29 (Nowell et al., 2016). Similarly, *P. savastanoi* strains encode 28 to 35 T3E genes (**Figure 6**). As expected, the only three core T3E genes of the *P. syringae* complex primary PGs (*avrE1, hopAA1*, and *hopAJ2*) and the broadly distributed *hopM1* (Dillon et al., 2019b), are also *P. savastanoi* core T3Es. *P. savastanoi* strains also encoded several T3E genes associated with pathogenicity on woody hosts, *i.e.* the core members *hopAO1*, *hopBL1,* and *hopBL2* (Matas et al., 2014; Nowell et al., 2016; Castañeda-Ojeda et al., 2017), as well as *hopAY1*, *hopH3*, *hopZ5,* and *hopAF1-1* (**Figure 6**). Additionally, all 20 genomes encode *hopAN1* and *hopJ1*, present in all 11 P*. syringae* complex PGs, but removed as T3Es or reclassified as T3SS helpers (Dillon et al., 2019b).

Truncations of T3E genes are common in *P. syringae* (Baltrus et al., 2011; Dillon et al., 2019a), and we considered them here (**Figure 7**) because some of their products are translocated into plant cells and interfere with plant defenses (Zumaquero et al., 2010; Matas et al., 2014). T3Es are considered the primary determinants of host specificity in the *P. syringae* complex at both the pathovar-species and race-cultivar levels (Collmer et al., 2000; Fouts et al., 2003; Cunnac et al., 2009; Lindeberg et al., 2009; Mansfield, 2009). We therefore searched for exclusive T3E genes present/absent in specific pathovars. All three Psr genomes encode *avrRps4*, *hopF1*, *hopZ5,* and the putative novel T3E gene B7R56_RS15315. Additionally, they lack *hopG1*, a gene found in the remaining 17 strains. These four known T3E genes were shown to contribute to the virulence of *P. syringae* strains but also function as avirulence factors in soybean and *Arabidopsis* (*avrRps4*), bean (*hopF1*), *Arabidopsis* (*hopZ5*) and cherry trees (*hopG1*) (Hinsch and Staskawicz, 1996; Tsiamis et al., 2000; Jayaraman et al., 2017; Hulin et al., 2018). Thus, *avrRps4*, *hopF1,* and *hopZ5* might contribute to Psr virulence in broom plants and/or restrict the infection of ash, oleander, and olive. Furthermore, Psr strains lack *hopG1* and *hopAF1-2*, or encode a truncated HopAF1-2 protein lacking its catalytic domain (**Figure 7**). Therefore, the absence of these T3Es could be related to their recognition by broom resistance genes.

Although we did not find Psn-exclusive T3E genes, Psn strains encode a specific 3′ truncation of *avrPto1* (**Figure 7**). Considering that point mutations affecting the C-terminal region of AvrPto abolish avirulence in tobacco (Shan et al., 2000), the specific Psn truncation might prevent avirulence in oleander. Other Psn-exclusive allelic variants possibly involved in host range definition are those of *hopAT1* and *hopA2* (**Figure 7**). Additionally, Psf and Psr strains do not encode a chimera of *avrRpm2*, with an N-terminal region homologous to that of the HopF4 family (Lo et al., 2017).

### Pathovar-Specific Genes

Using three complementary strategies, we identified a repertoire of pathovar-specific genes potentially associated with host specificity (**Figures 5**, **8**, and **Supplementary Table S2**). GView identified several genomic regions exclusively present/absent in all strains from monophyletic branches of the *P. savastanoi* phylogeny, most of which correspond to single pathovars (**Figure 8**). Since this strategy is not suitable for the identification of individual genes encoded by specific pathovars, we used the additional bioinformatics tools Roary (**Supplementary Table S2**) and PIFAR (**Figure 5**). Nevertheless, many of the pathovar-exclusive and pathovar-absent genes identified by Roary were also detected using GView or PIFAR, increasing the reliability of the results obtained.

As expected, all non-tumorigenic Psf strains lack the *iaaMH* operon (**Figure 5**), required for the biosynthesis of IAA and knot formation by Psv and Psn strains (Comai and Kosuge, 1983; Iacobellis et al., 1998; Aragón et al., 2014). Tumorigenic *P. savastanoi* pathovars, encode a copy of this operon in VR1, a genomic island also encoding the rhizobitoxine operon and two glutamine biosynthesis-related genes that likely supply glutamine for rhizobitoxine biosynthesis (Sugawara et al., 2006). In *Bradyrhizobium elkanii*, this enol-ether amino acid toxin enhances nodulation by inhibiting ethylene biosynthesis in host roots. Remarkably, production of IAA in *B. elkanii* is restricted to strains also synthetizing rhizobitoxine. Therefore, rhizobitoxine might also be involved in knot formation.

All pathovars, except Psn, contained genes for the biosynthesis of the phytotoxin phevamine A. This molecule, composed of a modified spermidine, l-phenylalanine, and l-valine, contributes to the virulence of *P. syringae* DC3000 on *Arabidopsis* suppressing immune responses induced by flagellin (O’Neill et al., 2018). We did not find genes for the biosynthesis of other phytotoxins involved in virulence in woody hosts (Hulin et al., 2018).

Gene *ptz*, involved in cytokinins production, was neither found in Psf strains nor in the genomes of Psn ICMP 13781 and the oleander strains here reassigned into Psv (0485_9 and ICMP 13786) (**Figure 5**). Gene *ptz* is plasmid encoded in most Psv and Psn strains, and is absent in some Psn strains (Pérez-Martínez et al., 2008). In addition, failure to produce cytokinins is correlated to symptoms attenuation, both on olive and oleander (Surico et al., 1985). However, Psn ICMP 13781 consistently induced tumors in olive as large as those produced by other Psn strains carrying gene *ptz* (**Figure 4**), suggesting that induction of full symptoms by Psn on olive is also dependent on other virulence determinants, as reported for Psv strains (Caballo-Ponce et al., 2017a).

In conclusion, besides T3SS genes whose role in host specificity has already been demonstrated in the *P. syringae* complex, our results suggest the existence of other putative virulence factors that could also contribute to host range definition, such as proteins secreted by the T2SS and T6SS, genes involved in the biosynthesis of the phytotoxins rhizobitoxine and phevamine A, a large number of transcriptional regulators, proteins involved in the metabolism of c-di-GMP, signal-transduction proteins and transporters of eight different superfamilies.

## Data Availability Statement

The datasets presented in this study can be found in online repositories. The names of the repository/repositories and accession number(s) can be found in the article/**Supplementary Material**.

## Author Contributions

JM, ST, CM, PR-P, and CR planned and designed the research and analyzed and interpreted the data. AM-P, AP and EC-P performed experiments. AM-P, AP, JM, PR-P, and CR carried out bioinformatics analyses. AM-P, AP, JM, and CR designed and prepared Figures and Tables. AM-P, AP, EC-P, JM, and CR wrote the manuscript.

## Funding

AM-P, AP, CR, and JM were supported by grants FPI/BES-2015-074847, FPU14/05551, AGL2017-82492-C2-1-R and AGL2017-82492-C2-2-R, respectively, from *Ministerio de Ciencia, Innovación y Universidades* (Spain), cofinanced by the *Fondo Europeo de Desarrollo Regional* (FEDER); CM was supported by DSA3 research funds “Fondo di base” (Italy).

## Conflict of Interest

The authors declare that the research was conducted in the absence of any commercial or financial relationships that could be construed as a potential conflict of interest.

## Footnotes

1
https://www.ncbi.nlm.nih.gov/

2
http://usegalaxy.org/

3
http://effectivedb.org

4
https://software.broadinstitute.org/morpheus/

5
https://server.gview.ca/

6
http://www.pseudomonas-syringae.org/

7
https://pfam.xfam.org/

8
https://toolkit.tuebingen.mpg.de/tools/hhpred
