# Supplementary material for: Host Range Determinants of Pseudomonas savastanoi Pathovars of Woody Hosts Revealed by Comparative Genomics and Cross-Pathogenicity Tests
Source: Front Plant Sci. 2020 Jul 2;11:973. doi: 10.3389/fpls.2020.00973 (PMC7343908; doi:10.3389/fpls.2020.00973)
Supplement: Supplementary file 1 [file DataSheet_1.docx]

**Supplementary Figure S1.** Gene content of selected variable regions present or absent in specific *P. savastanoi* pathovars or strains. The indicated gene identification (ID) numbers correspond to *P. savastanoi* pv. savastanoi NCPPB 3335. Black, white and grey boxes indicate presence, absence, or truncation, respectively, of the corresponding gene in the indicated genomes. Psv, Psn, Psf and Psr, *P. savastanoi* pathovars savastanoi, nerii, fraxini and retacarpa, respectively. Asterisks indicate oleander strains included in pathovar Psv (see Figure 4).

**Supplementary Figure S1.** Continuation.


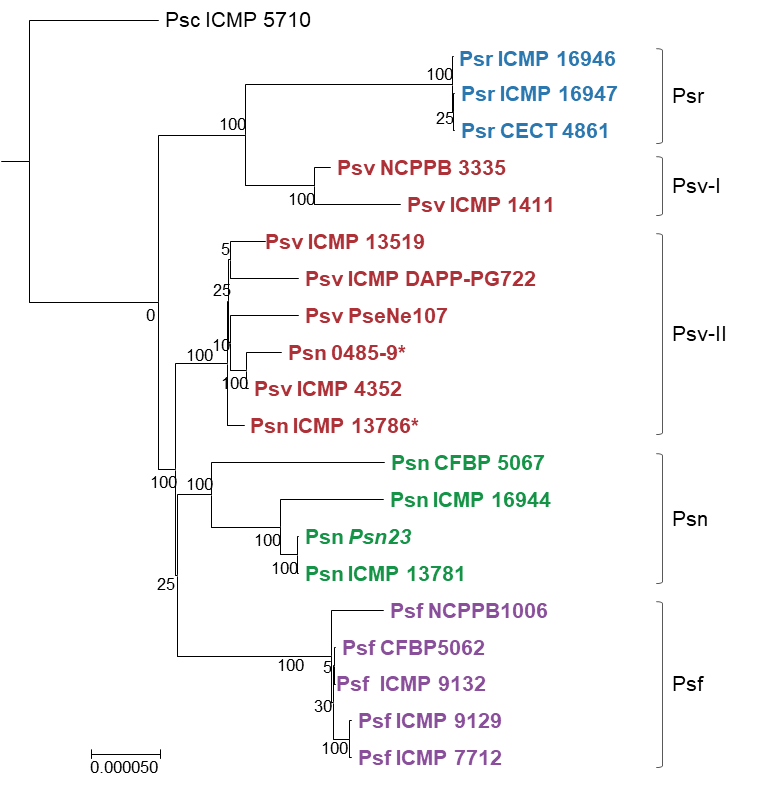


**Supplementary Figure S2.** MLSA phylogeny of *P. savastanoi* pathovars. Forty genes whose sequences are complete in all the assemblies (Supplementary Table S1) were concatenated (80,902 to 80,940 nt per strain) and aligned using Muscle with MEGA 7. The alignment was manually curated and used to construct a tree using RaxML 7.2.8 within Geneious 8.1.9. All positions and gaps were used with a GTR gamma model. The tree was rooted using the genome of *P. syringae* pv. ciccaronei ICMP 5710 (assembly LJPY01). Values in nodes are bootstrap percentages of 200 iterations. *P. savastanoi* pathovars abbreviations as in Supplementary Figure S1. Asterisks indicate oleander strains included in pathovar Psv (see Figure 4).


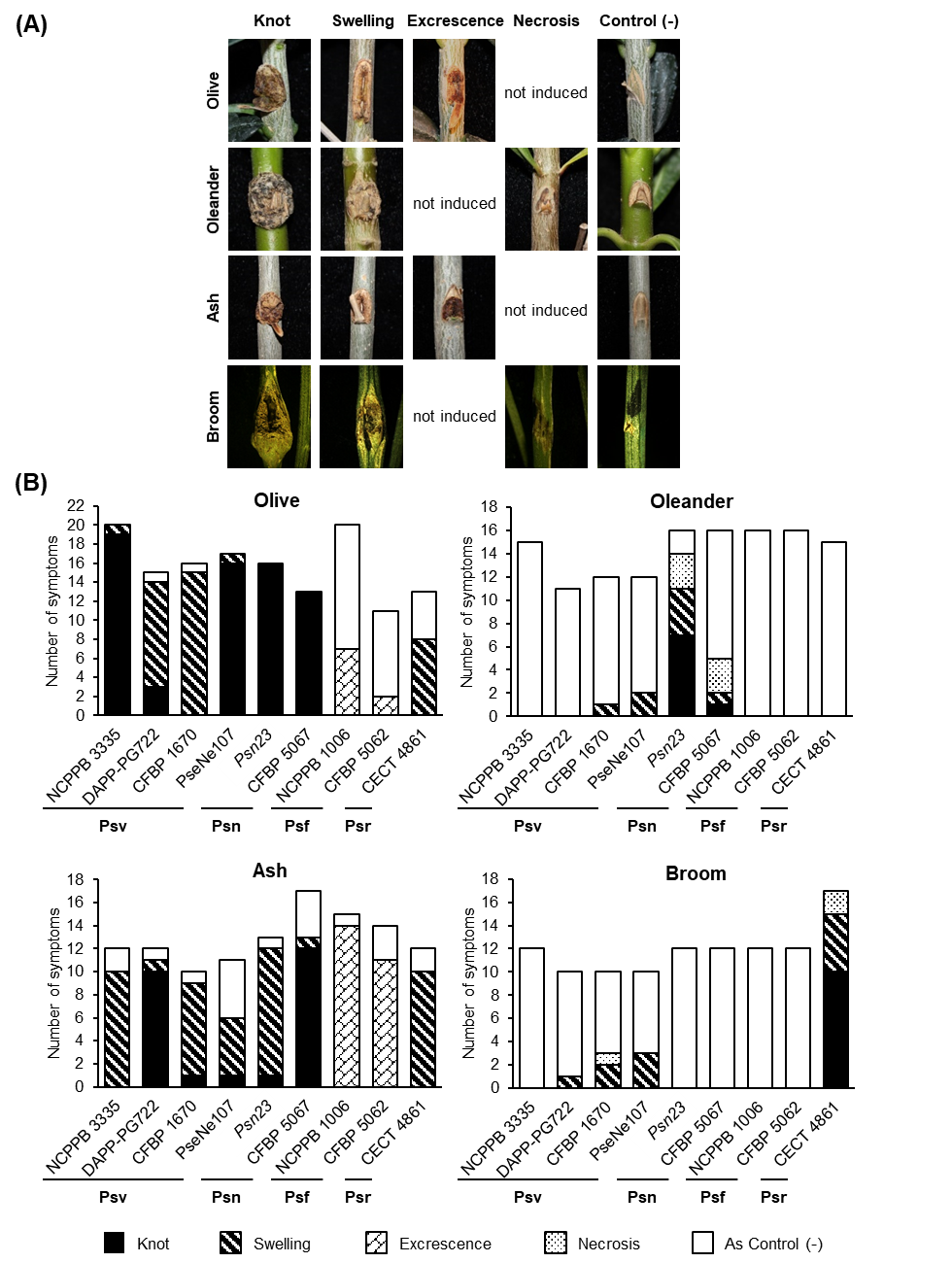


**Supplementary Figure S3.** Cross-pathogenicity tests of *P. savastanoi* strains on diverse woody hosts. (A) Diversity of symptoms generated on olive, oleander, ash and broom. Control (-), plants inoculated with 10 mM MgCl_2_. Symptoms were recorded at 90 days post-inoculation. (B) Number of inoculation points showing each of the different symptoms generated by *P. savastanoi* strains on each plant host. Psv ICMP 4352 was obtained from the CFBP collection as strain CFBP 1670. Strain abbreviations as in Supplementary Figure S1.


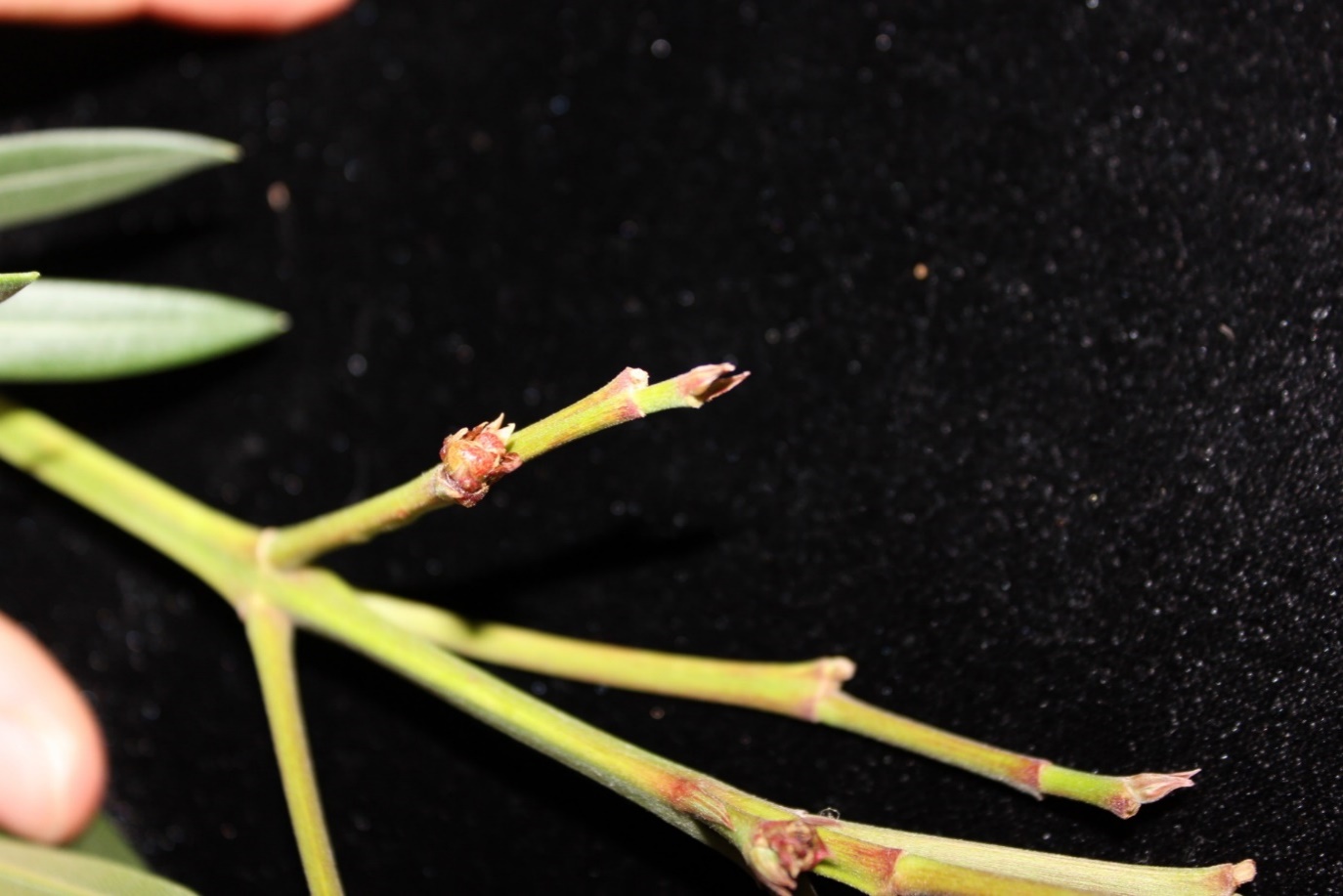

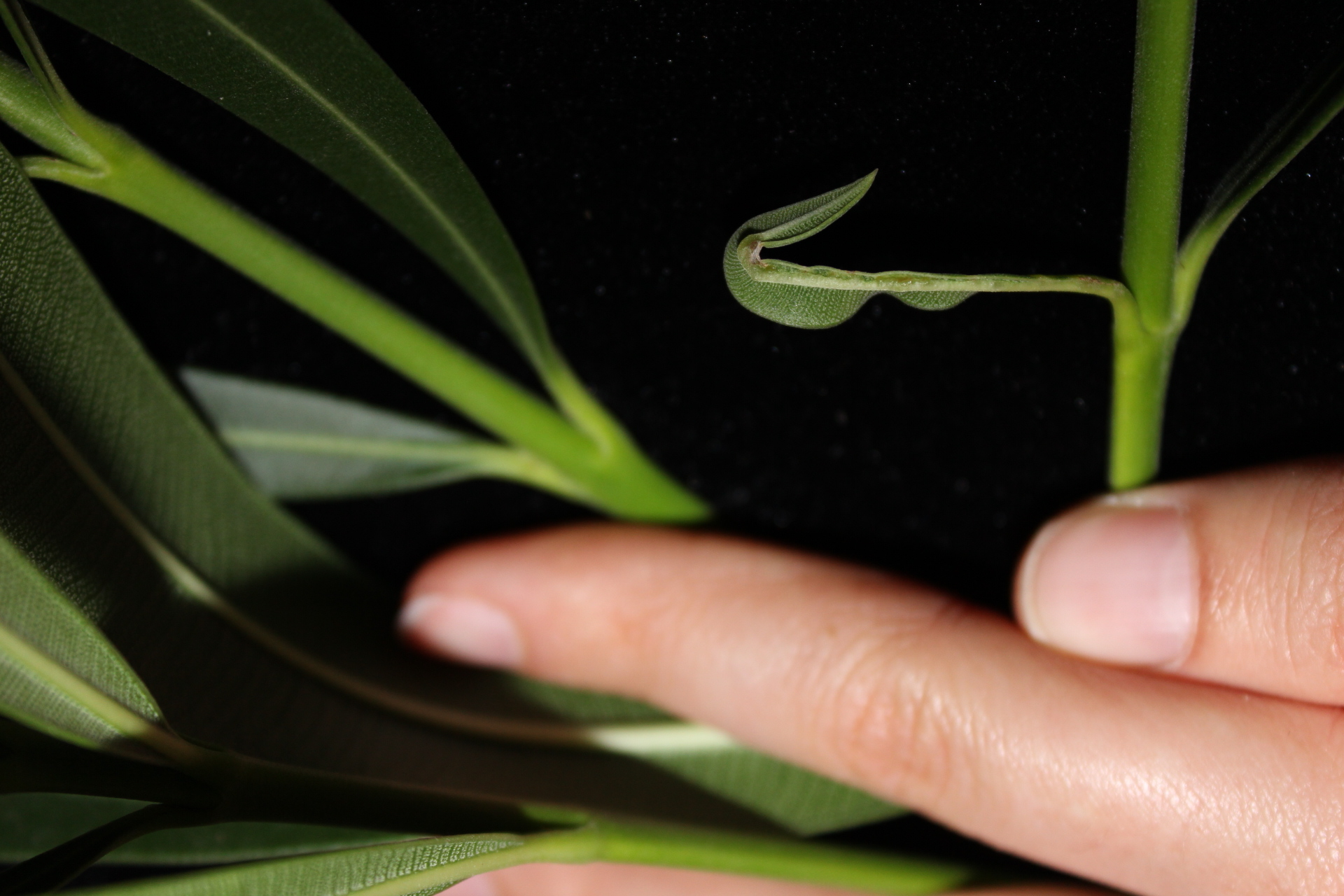

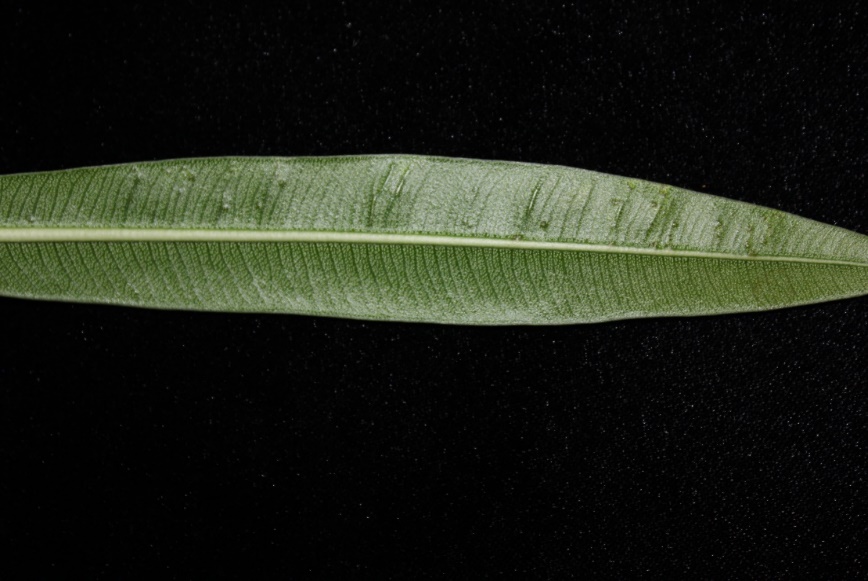


**(A)**

**(C)**

**(B)**

**Supplementary Figure S4.** Development of secondary symptoms caused by *P. savastanoi* pv. nerii CFBP 5067 on oleander plants. Symptoms were recorded at 90 days post-inoculation. **(A)** Flower bud swelling, **(B)** formation of small knots on the leaves, and **(C)** curled leaf.
